# Supplementary material for: Bioinformatics Approach for Identifying Novel Biomarkers and Their Signaling Pathways Involved in Interstitial Cystitis/Bladder Pain Syndrome with Hunner Lesion
Source: J Clin Med. 2020 Jun 21;9(6):1935. doi: 10.3390/jcm9061935 (PMC7356914; doi:10.3390/jcm9061935)
Supplement: Supplementary file 1 [file jcm-09-01935-s001.pdf]

Table S1. Sample information of each dataset used in this study

GSE28242			
**Disease State**	**Age**	**Gender**	**Ulceration**
Non-IC/BPS (n = 5)	51	F	No
	51	F	No
	38	M	No
	71	F	No
	75	F	No
IC/BPS (n = 3)	82	M	Yes
	76	F	Yes
	55	F	Yes

GSE11783			
**Disease State**	**Age**	**Gender**	**Ulceration**
Non-IC/BPS (n = 6)	59.2 (40.0–75.0)	F	No
		F	No
		F	No
		F	No
		F	No
		F	No
IC/BPS (n = 5)	78 (68.0–85.0)	F	Yes
		F	Yes
		F	Yes
		F	Yes
		F	Yes

GSE57560					
**Disease State**	**Age**	**Gender**	**Glomerulation**	**Ulceration**	**Cystectomy**
Non-IC/BPS (n = 3)	76	F	NA	No	No
	43	F	NA	No	No
	53	F	NA	No	No
IC/BPS (n = 2)	67	F	Severe	Yes	Yes
	65	F	Mild	Yes	Yes

Table S2. Fifty-three DEGs (up- and downregulated genes) with *p*-values and FC.

	GSE28242		GSE11783		GSE57560	
Gene	*p*-Value	LogFC	*p*-Value	LogFC	*p*-Value	LogFC
ABCD2	2.80E-02	1.13	3.20E-03	1.69	2.30E-04	2.08
ADCY7	1.70E-02	1.13	3.90E-04	1.35	6.10E-03	1.79
BTLA	2.80E-02	1.18	3.60E-03	1.25	2.80E-02	2.99
CD180	4.00E-03	1.12	8.90E-05	2.38	2.70E-02	2.68
CD38	4.80E-02	1.48	8.50E-07	4.33	1.90E-03	4.31
CD5	3.00E-02	1.00	9.00E-04	1.63	6.90E-03	3.49
CD84	3.30E-02	1.32	4.80E-04	1.09	1.20E-02	1.33
CFB	8.60E-03	2.08	1.10E-06	2.61	4.20E-02	2.90
CLIC2	1.50E-02	1.63	2.20E-04	1.22	3.50E-03	1.36
CTSZ	8.10E-03	1.02	2.90E-03	1.11	7.00E-03	1.13
CYSLTR1	2.40E-02	2.30	3.70E-05	1.54	2.60E-02	1.26
DOCK10	1.20E-02	1.54	4.20E-04	1.79	1.20E-02	2.01
EPSTI1	5.50E-04	2.50	5.50E-03	1.14	1.50E-03	2.27
FYN	2.10E-02	1.46	2.90E-04	1.29	5.30E-03	1.73
GIMAP4	2.60E-02	1.95	2.10E-03	1.06	1.70E-03	2.02
GIMAP8	2.90E-04	1.01	1.10E-03	1.14	2.60E-02	1.17
GLRX	2.90E-02	1.68	3.50E-04	1.15	2.70E-02	1.31
GPR171	7.20E-03	1.52	3.70E-05	1.71	2.10E-03	3.36
GPRIN3	6.10E-03	1.03	8.20E-06	1.70	4.20E-04	2.60
GZMK	6.30E-03	1.50	1.60E-02	1.64	8.00E-03	3.10
HLA-DMA	1.80E-02	1.83	2.80E-04	1.25	1.80E-03	2.49
HLA-DMB	4.40E-03	1.92	3.40E-03	1.08	5.50E-03	2.72
HLA-DOB	4.90E-02	1.31	1.20E-03	2.97	3.00E-03	3.10
HLA-DPA1	2.90E-02	1.76	1.20E-02	1.54	4.50E-03	2.32
IDO1	2.70E-02	3.58	4.30E-03	2.03	1.70E-05	4.69
IFI27	3.00E-02	1.51	1.40E-04	1.69	1.70E-03	2.07
IFITM3	1.80E-02	1.57	7.20E-04	1.14	2.30E-02	1.29
IL32	2.00E-03	1.45	2.40E-03	1.34	5.90E-03	2.55
IL7R	5.00E-02	1.33	5.30E-03	1.49	4.10E-03	2.69
ITGA4	1.80E-03	2.10	6.50E-05	1.36	3.40E-04	1.69
ITGAL	4.50E-02	1.72	3.40E-03	1.23	6.90E-03	2.40
ITK	2.90E-02	1.42	4.80E-04	1.50	1.40E-02	3.18
KCNA3	4.20E-02	1.34	1.80E-03	3.22	1.20E-02	2.21
KLRB1	3.00E-02	1.13	1.20E-03	1.49	1.20E-02	2.57
LEF1	8.50E-03	1.06	5.10E-03	1.11	1.40E-02	2.19
MCOLN2	3.80E-02	1.13	1.30E-02	1.30	8.00E-03	2.55
NCKAP1L	2.10E-02	1.93	3.30E-05	1.65	5.70E-03	1.97
NLRC5	1.30E-02	1.78	2.20E-04	1.56	4.40E-02	1.70
PLAC8	4.20E-03	1.46	1.10E-04	1.99	5.40E-03	4.22
PSMB9	1.20E-02	2.40	2.30E-03	1.15	3.30E-04	2.42
RASGRP1	3.70E-02	1.06	3.50E-05	1.86	6.40E-03	2.65
SAMHD1	6.60E-04	1.46	1.40E-05	1.87	2.40E-03	1.78
SEPT6	2.20E-02	1.19	3.60E-05	1.57	1.10E-02	1.32
SH2D1A	1.20E-02	1.21	9.90E-04	1.95	2.10E-03	3.11
SLAMF8	2.20E-02	1.01	1.60E-03	1.69	2.00E-03	3.00
SLFN11	2.20E-03	1.25	3.10E-04	1.17	4.30E-03	1.42
STAT1	1.40E-02	1.85	2.90E-02	1.46	6.40E-03	2.91
TAP2	2.40E-02	2.13	4.60E-04	1.46	4.30E-04	1.61
TGM2	1.30E-03	2.53	5.70E-05	1.64	1.60E-03	1.60
TRAT1	1.10E-02	1.43	1.60E-03	1.71	4.60E-03	3.04
WARS	1.80E-02	2.14	3.30E-04	1.45	3.70E-03	2.41
CWH43	3.00E-02	−1.45	9.50E-07	−5.58	1.80E-04	−5.92
CYP2J2	4.10E-02	−1.02	6.80E-06	−4.72	1.00E-02	−1.52
